# Olfactory ensheathing glia are required for embryonic olfactory axon targeting and the migration of gonadotropin-releasing hormone neurons

**DOI:** 10.1242/bio.20135249

**Published:** 2013-06-21

**Authors:** Perrine Barraud, James A. St John, C. Claus Stolt, Michael Wegner, Clare V. H. Baker

**Affiliations:** 1 Department of Physiology, Development and Neuroscience, University of Cambridge, Cambridge CB2 3DY, UK; 2 Eskitis Institute for Drug Discovery, Griffith University, Brisbane QLD 4111, Australia; 3 Institut für Biochemirich-Alexander-Universität Erlangen, 91054 Erlangene, Emil-Fischer-Zentrum, Fried, Germany

**Keywords:** Sox10, Olfactory ensheathing glia, GnRH neurons, Kallmann's syndrome

## Abstract

Kallmann's syndrome is caused by the failure of olfactory axons and gonadotropin-releasing hormone (GnRH) neurons to enter the embryonic forebrain, resulting in anosmia and sterility. *Sox10* mutations have been associated with Kallmann's syndrome phenotypes, but their effect on olfactory system development is unknown. We recently showed that *Sox10* is expressed by neural crest-derived olfactory ensheathing cells (OECs). Here, we demonstrate that in homozygous *Sox10^lacZ/lacZ^* mouse embryos, OEC differentiation is disrupted; olfactory axons accumulate in the ventromedial olfactory nerve layer and fewer olfactory receptor neurons express the maturation marker OMP (most likely owing to the failure of axonal targeting). Furthermore, GnRH neurons clump together in the periphery and a smaller proportion enters the forebrain. Our data suggest that human *Sox10* mutations cause Kallmann's syndrome by disrupting the differentiation of OECs, which promote embryonic olfactory axon targeting and hence olfactory receptor neuron maturation, and GnRH neuron migration to the forebrain.

## Introduction

The anosmia and sterility of Kallmann's syndrome arise when olfactory axons and gonadotropin-releasing hormone (GnRH) neurons, which are needed for pituitary gonadotropin release, fail to enter the embryonic forebrain (Cadman et al., 2007; Cariboni et al., 2007; Hardelin and Dodé, 2008). GnRH neurons migrate from the embryonic olfactory epithelium along olfactory and/or vomeronasal nerves into the forebrain (Cariboni et al., 2007; Wray, 2010; Wierman et al., 2011). Recently, spontaneous mutations in the transcription factor gene *Sox10* were associated with Kallmann's syndrome phenotypes: anosmia, hypogonadism and cryptorchidism (Bondurand et al., 2007; Barnett et al., 2009). *Sox10* is expressed by migrating neural crest cells and required for the specification and differentiation of neural crest-derived Schwann cells and satellite glia (Herbarth et al., 1998; Southard-Smith et al., 1998; Britsch et al., 2001; Paratore et al., 2002; Finzsch et al., 2010). We recently showed that olfactory ensheathing cells (OECs), which ensheath olfactory axons from the epithelium to their targets in the olfactory bulb (Ekberg et al., 2012), are neural crest-derived and express *Sox10* (Barraud et al., 2010). Sox10 expression was subsequently reported in mouse OECs from E10.5 (Forni et al., 2011), when olfactory axons and migratory neurons first emerge from the olfactory epithelium (Valverde et al., 1992; Miller et al., 2010). Here, we test the hypothesis arising from the association of *Sox10* mutations with Kallmann's syndrome, namely that *Sox10* is required for OEC differentiation and that OECs are required for the entry of olfactory axons and GnRH neurons into the embryonic forebrain.

## Materials and Methods

### Embryo collection and sectioning

*Sox10^lacZ^* mutant mice (Britsch et al., 2001) and wild-type litter-mates of C3HeB/FeJ background were obtained from heterozygous crosses. Embryos were immersion-fixed overnight in 4% paraformaldehyde in phosphate-buffered saline (PBS) at 4°C. Genotypes were determined from tail biopsies as described (Britsch et al., 2001). Embryos were embedded for wax or cryosectioning and sectioned at 5–6 µm (or at 30 µm, for some E16.5 embryos).

### Immunohistochemistry

Immunohistochemistry was performed as described (Lassiter et al., 2007). Primary antibodies used were: anti-β galactosidase (chicken, Abcam; 1:1000); anti-BLBP (rabbit, Millipore; 1:1000), anti-GnRH-1 (rabbit, Abcam; 1:100), anti-HuC/D (mouse IgG2b, Invitrogen; 1:500), anti-laminin (rabbit, Sigma; 1:1000), anti-NCAM (rabbit, Millipore, 2 µg/ml); anti-neuronal βIII tubulin (Tuj1, mouse IgG2a, Covance; 1:500), anti-neuronal βIII tubulin (rabbit, Abcam, 1:1000), anti-NPY (rabbit, Abcam, 1:6000), anti-OMP (goat, Wako; 1:500 or 1:1000), anti-p75^NTR^ (rabbit, kind gift of L. Reichardt, University of California at San Francisco, USA; 1:1000), anti-S100 (rabbit, DAKO; 1:50), anti-Sox10 (goat, Santa Cruz Biotechnology; 1:100). Appropriately matched Alexa Fluor 488-, 568- or 594-conjugated secondary antibodies, Alexa Fluor 350-NeutrAvidin and Alexa Fluor 488-streptavidin were obtained from Invitrogen, and biotinylated secondary antibodies from Southern Biotech.

### In situ hybridization

Primers against mouse *GnRH1* (GenBank accession number NM_008145.2) were designed using Primer3 Input (Rozen and Skaletsky, 2000). Total RNA was extracted from the snout and part of the forebrain using Trizol (Invitrogen), and single-strand cDNA generated using Invitrogen's Superscript III First-Strand Synthesis System kit. *GnRH1* was amplified by PCR (forward primer: CTCAACCTACCAACGGAAGC; reverse primer: GGGCCAGTGCATCTACATCT). The 344 bp product was cloned into pDrive (Qiagen) using the Qiagen PCR Cloning Kit and sequenced (Biochemistry Department DNA Sequencing Facility, Cambridge, UK). Digoxigenin-labelled antisense riboprobes were generated (Henrique et al., 1995) and in situ hybridization performed on sections as described (Xu et al., 2008).

### Statistical analysis of olfactory receptor neuron maturation and olfactory epithelium thickness

Confocal images covering an optical depth of 15 µm were captured from 30 µm sections through the olfactory mucosa of E16.5 embryos (two wild-type, two *Sox10^lacZ/+^* and three *Sox10^lacZ/lacZ^* embryos). Adjacent sections were immunostained for OMP and neuronal βIII tubulin. The region of interest covered a 200 µm length of the nasal septum in the middle portion of the dorsal–ventral span of the olfactory mucosa. Three sections were quantified/embryo for each marker, with each section being 240 µm apart (480 µm total rostral–caudal distance); the first section was 300 µm from the most rostral portion of the olfactory bulb. All cells expressing OMP or neuronal βIII tubulin within the imaged regions of interest were counted. For each of the three sections quantified/embryo, the number of OMP-positive and neuronal βIII tubulin-positive cells within the olfactory epithelium on each side of the nasal septum was counted (i.e., 6 measurements/embryo for each marker), and the thickness of the epithelium (from the nasal surface to the basal lamina) measured at three different positions on each side of the septum (i.e., 18 measurements per embryo). The mean/embryo was determined for each measurement, which was converted from pixels to µm and presented as OMP-positive or neuronal βIII tubulin-positive cell count/100 µm of olfactory epithelium, or thickness of olfactory epithelium in µm. GraphPad Prism (GraphPad Software, La Jolla, California, USA) was used to perform one-way ANOVA using Tukey's multiple comparison test (comparing every mean with every other mean) and unpaired 2-tailed t-tests.

### Statistical analysis of GnRH neuron distribution

GnRH1 neurons were counted on 5–6 µm serial sections (10 slides/series: on each slide, each section was collected every 50–60 µm) processed for immunohistochemistry or in situ hybridization to detect GnRH1. At least 100 GnRH1 neurons/embryo were counted on serial parasagittal sections of E14.5 embryos from three different litters (3 wild-type, 3 heterozygous *Sox10^lacZ/+^* embryos, 3 homozygous *Sox10^lacZ/lacZ^* embryos) and on serial coronal sections of E16.5 embryos from four different litters (4 wild-type, 4 heterozygous *Sox10^lacZ/+^* embryos, 4 homozygous *Sox10^lacZ/lacZ^* embryos). Differences between the means for groups of the same stage (wild-type versus heterozygous *Sox10^lacZ/+^* embryos, and wild-type versus homozygous *Sox10^lacZ/lacZ^* embryos) were assessed via one-way ANOVA using Dunnett's multiple comparison test, performed using GraphPad Prism (GraphPad Software, La Jolla, California, USA).

## Results and Discussion

### Sox10 expression in the developing olfactory system is restricted to OECs (and, at later stages, Bowman's gland/duct cells)

**Fig. 1.**
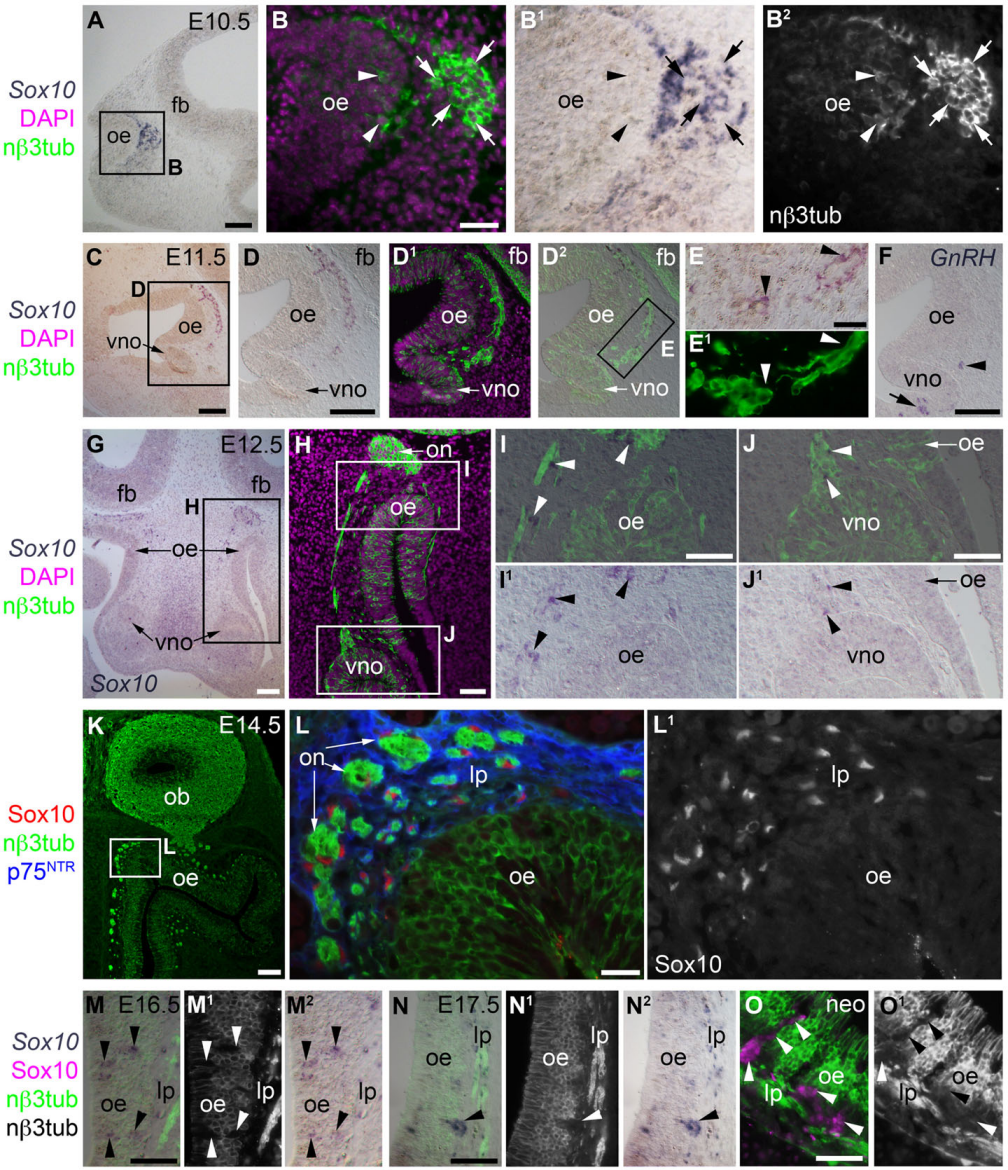
During mouse olfactory system development, Sox10 expression is restricted to OECs (and, at late stages, to Bowman's gland/duct cells). (**A–B^2^**) At E10.5, in situ hybridization (ISH) on parasagittal sections followed by immunostaining for neuronal βIII tubulin (nβ3tub) reveals *Sox10* expression in non-neuronal cells associated with the “migratory mass” of olfactory axons and migrating neurons emerging from the olfactory epithelium. *Sox10* is not expressed by neurons in either the olfactory epithelium or the migratory mass (examples of each are indicated, respectively, by arrowheads and arrows). (**C–F**) At E11.5, when the developing vomeronasal organ evaginates from the ventromedial olfactory epithelium (Cuschieri and Bannister, 1975), ISH shows (C–E^1^) *Sox10* expression in non-neuronal cells (OECs; arrowheads in E,E^1^) associated with olfactory and vomeronasal (adjacent to the vomeronasal organ) nerves, but no *Sox10* expression in the neurons within either the olfactory or vomeronasal epithelia; and (F) *GnRH1*-positive cells within the vomeronasal organ epithelium (arrow) and in the region of the vomeronasal nerve (arrowhead; compare with position of neurons in D^1^–E^1^). (**G–J^1^**) At E12.5, ISH on coronal sections followed by immunostaining for nβ3tub shows *Sox10* expression in non-neuronal cells associated with the olfactory and vomeronasal nerves, but no above-background *Sox10* expression within either the olfactory or vomeronasal epithelia. (**K–L^1^**) At E14.5, immunostaining on coronal sections reveals Sox10-positive, p75^NTR^-positive OECs surrounding olfactory nerve fascicles in the lamina propria, but no Sox10 expression in neurons in the olfactory epithelium. (**M–N^2^**) At E16.5 (M–M^2^) and E17.5 (N–N^2^), ISH for *Sox10* on coronal sections followed by immunostaining for nβ3tub shows non-neuronal *Sox10*-positive cells in the olfactory epithelium (arrowheads): these are developing Bowman's gland/duct cells, which begin to protrude from the basal epithelium from E17.5 (Cuschieri and Bannister, 1975). (**O**,**O^1^**) In neonates, immunostaining for Sox10 and nβ3tub on coronal sections shows that Bowman's gland/duct cells (arrowheads) maintain Sox10 expression after birth. Abbreviations: fb, forebrain; lp, lamina propria; nβ3tub, neuronal βIII tubulin; neo, neonatal; ob, olfactory bulb; oe, olfactory epithelium; on, olfactory nerve; vno, vomeronasal organ. Scale bars: 100 µm (C,D,F,G,K), 50 µm (A,H,I,J,M,N,O), 20 µm (B,L), 10 µm (E).

We aimed to understand how Kallmann's syndrome phenotypes could result from *Sox10* mutations (Bondurand et al., 2007; Barnett et al., 2009). We used in situ hybridization (ISH) and immunostaining to examine Sox10 expression during mouse olfactory system development from E10.5 to neonatal stages (Fig. 1A–O^1^). Our results confirm and extend previous reports (Barraud et al., 2010; Forni et al., 2011) showing that Sox10 expression is restricted to OECs (which are found along the entire length of the olfactory nerve throughout its development), apart from Bowman's gland/duct cells in the olfactory epithelium at later stages (as we previously described for avian embryos; Barraud et al., 2010). Sox10 expression was not seen (by either ISH or immunostaining) in neurons in the olfactory epithelium at any stage examined (Fig. 1A–O^1^). Likewise, *Sox10* expression was not seen in neurons in the vomeronasal organ epithelium (e.g. Fig. 1C–D^2^,G–J^1^), or in the neurons (which include GnRH neurons) migrating along olfactory and/or vomeronasal nerves (e.g. Fig. 1C–J^1^). At E16.5, non-neuronal *Sox10*-positive cells were clearly visible within the olfactory epithelium (Fig. 1M–M^2^). From E17.5 until at least neonatal stages, these were found in large clusters protruding into the mesenchyme (Fig. 1N–O^1^), and as strands projecting across the width of the epithelium (Fig. 1O,O^1^). As we previously reported for avian embryos [figure S8 in Barraud et al. (Barraud et al., 2010)], these non-neuronal Sox10-positive cells in the olfactory epithelium can be identified as developing Bowman's gland/duct cells, which start to protrude from the mouse olfactory epithelium at E17.5 (Cuschieri and Bannister, 1975). Overall, therefore, while GnRH neurons are migrating (Cariboni et al., 2007) and olfactory axons reach the olfactory bulb, Sox10 expression is restricted to OECs during mouse olfactory system development.

### OEC differentiation is disrupted after *Sox10* deletion

**Fig. 2.**
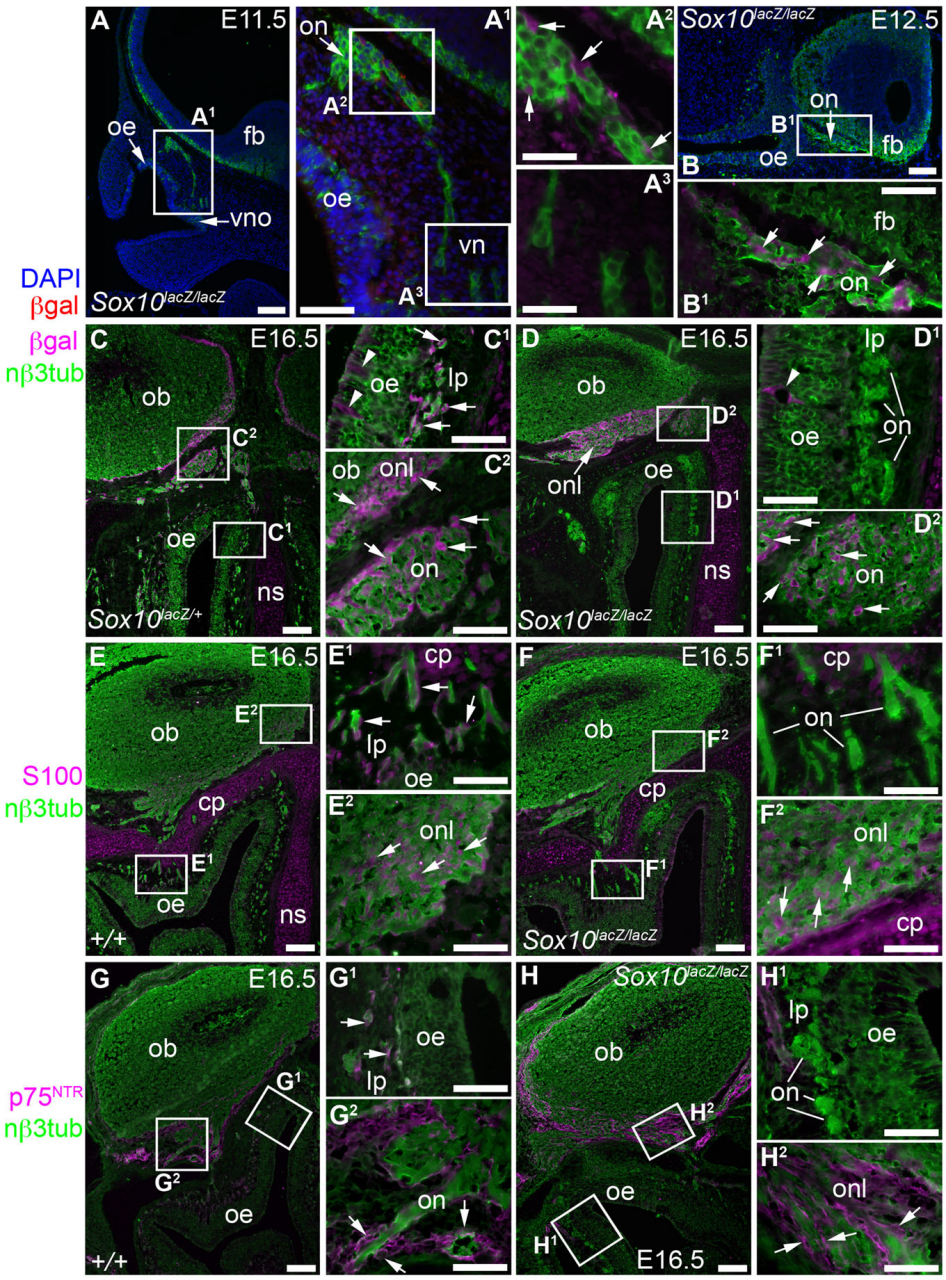
After *Sox10* deletion, neural crest cells colonize the olfactory nerve but are missing from the lamina propria by E16.5. (**A–B^1^**) In homozygous *Sox10^lacZ/lacZ^* embryos at E11.5 (A–A^3^) and E12.5 (B,B^1^), immunostaining on parasagittal sections reveals β-gal-positive cells associated with the olfactory nerve (arrows, A^2^,B^1^). β-gal expression in mesenchymal cells beneath the olfactory epithelium (A^1^) may reflect protein perdurance in neural crest-derived cells. (**C–D^2^**) At E16.5, immunostaining on coronal sections shows β-gal-positive cells (arrows) throughout the olfactory nerve from the lamina propria to the ONL in heterozygous *Sox10^lacZ/+^* embryos (C–C^2^), but absent from the lamina propria after *Sox10* deletion (D–D^2^). Arrowheads in C^1^,D^1^ indicate β-gal-positive prospective Bowman's gland/duct cells in the epithelium, which express Sox10 (Fig. 1N–O^1^). Fainter β-gal immunoreactivity in cribriform plate and nasal septum cartilage (C,C^1^,D,D^1^) may be specific: weak β-gal staining was previously reported in limb cartilage condensations in *Sox10^lacZ^* embryos (Britsch et al., 2001). (**E–F^2^**) At E16.5, immunostaining on coronal sections reveals S100 expression (arrows) throughout the olfactory nerve from the lamina propria to the ONL in wild-type embryos (E–E^2^), but absent from the lamina propria after *Sox10* deletion (F–F^2^). (**G–H^2^**) At E16.5, immunostaining on coronal sections shows p75^NTR^ expression (arrows) throughout the olfactory nerve from the lamina propria to the ONL in wild-type embryos (G–G^2^), but absent from olfactory nerve fascicles in the lamina propria after *Sox10* deletion (H–H^2^). Abbreviations: βgal, β-galactosidase; cp, cribriform plate; fb, forebrain; lp, lamina propria; nβ3tub, neuronal βIII tubulin; ns, nasal septum; ob, olfactory bulb; oe, olfactory epithelium; on, olfactory nerve; onl, olfactory nerve layer; vn, vomeronasal nerve; vno, vomeronasal organ. Scale bars: 100 µm (A,B,C,D,E,F,G,H), 50 µm (A^1^,B^1^,C^1^,C^2^,D^1^,D^2^,E^1^,E^2^,F^1^,F^2^,G^1^,G^2^,H^1^,H^2^), 25 µm (A^2^,A^3^).

**Fig. 3.**
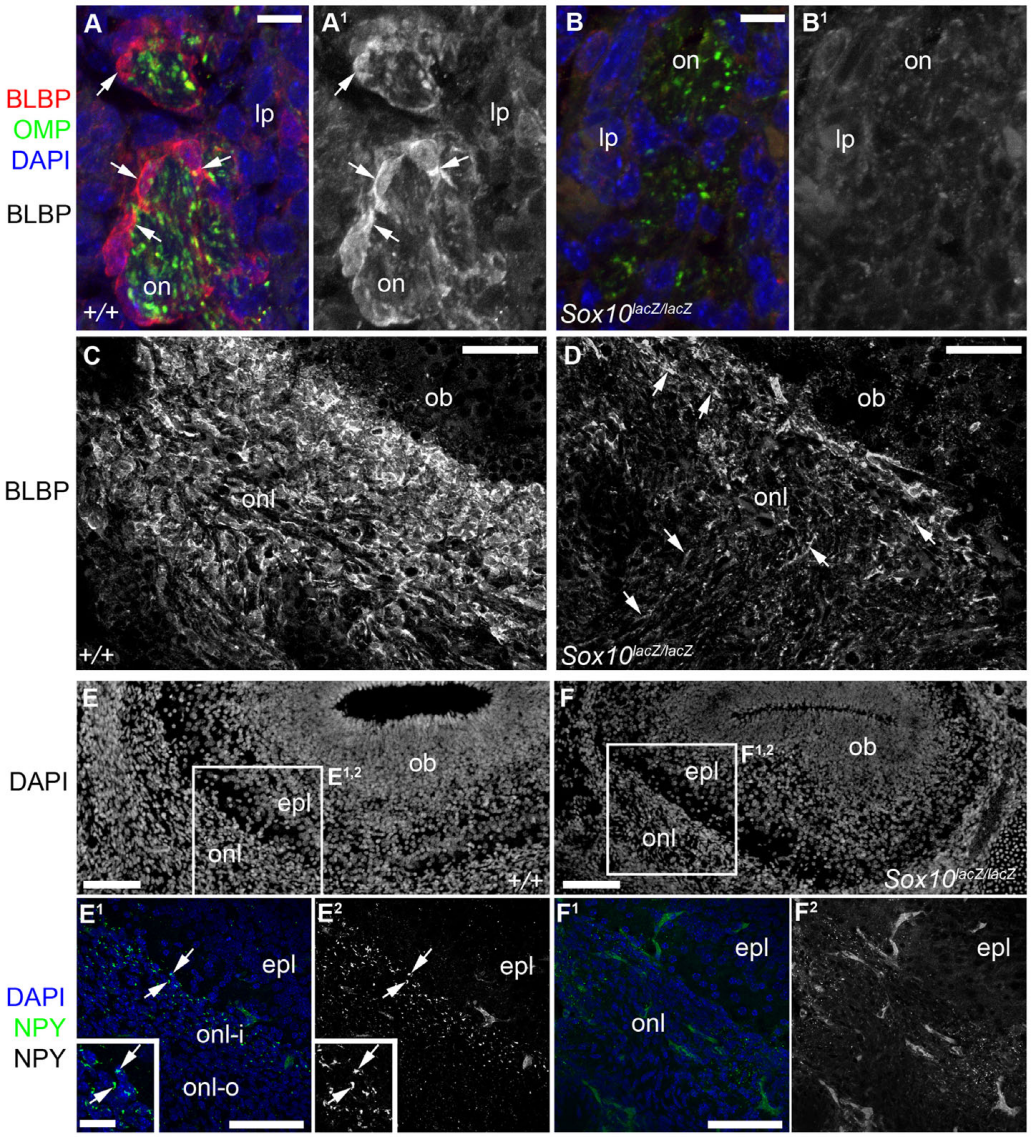
After *Sox10* deletion, OEC differentiation is defective. All images show E16.5 coronal sections. (**A–B^1^**) Expression of the early glial differentiation marker BLBP is seen in OECs ensheathing olfactory nerve fascicles (labelled by immunostaining for olfactory marker protein) in the wild-type lamina propria (A,A^1^), but not after *Sox10* deletion (B,B^1^). (**C**,**D**) BLBP expression in the ONL is much stronger in wild-type (C) than homozygous *Sox10^lacZ/lacZ^* embryos (D). (**E–F^2^**) At E16.5, NPY expression is seen in the inner ONL of wild-type embryos (arrows, E^1^,E^2^) but undetectable after *Sox10* deletion (F^1^,F^2^). Abbreviations: epl, external plexiform layer; lp, lamina propria; ob, olfactory bulb; OMP, olfactory marker protein; on, olfactory nerve; onl, olfactory nerve layer; onl-i, inner olfactory nerve layer; onl-o, outer olfactory nerve layer. Scale bars: 100 µm (E,E^1^,F,F^1^), 50 µm (C,D), 25 µm (E^1^ inset), 10 µm (A,B).

We examined OEC and olfactory system development in mouse embryos in which the *Sox10* open reading frame was replaced by *lacZ* (Britsch et al., 2001). We could identify surviving *Sox10*-mutant cells by immunostaining for β-galactosidase (β-gal). As reported for other peripheral nerves (Britsch et al., 2001; Paratore et al., 2002), neural crest-derived cells colonized the developing olfactory nerve even after *Sox10* deletion, since at E10.5–E12.5, at least some β-gal-expressing cells were present in the olfactory nerve in homozygous *Sox10^lacZ/lacZ^* embryos (*n* = 3; Fig. 2A–B^1^). By E16.5, β-gal-expressing cells in heterozygous *Sox10^lacZ/+^* embryos were distributed along the olfactory nerve from the lamina propria to the ONL (*n* = 3; Fig. 2C–C^2^). In contrast, β-gal-expressing cells were missing from the lamina propria at E16.5 after *Sox10* deletion (*n* = 3; Fig. 2D,D^1^), though present on the proximal part of the olfactory nerve, near its entry-point into the olfactory bulb, and within the ONL (Fig. 2D,D^2^). Similarly, immunoreactivity for S100 (Fig. 2E–F^2^) and p75^NTR^ (Fig. 2G–H^2^) was absent from the lamina propria after *Sox10* deletion (S100, Fig. 2F,F^1^; p75^NTR^, Fig. 2G,G^1^), though present in the proximal olfactory nerve and ONL (S100, Fig. 2F,F^2^; p75^NTR^, Fig. 2G,G^2^). However, p75^NTR^ is also expressed by undifferentiated neural crest cells in rodents (Stemple and Anderson, 1992; Rao and Anderson, 1997), and expression of the early glial differentiation marker brain lipid binding protein (BLBP; Fig. 3A–D) (Murdoch and Roskams, 2007), which was also absent from the lamina propria after *Sox10* deletion (Fig. 3B,B^1^), was significantly weaker in the proximal olfactory nerve and ONL (Fig. 3D). This suggests that a glial specification/differentiation defect affects most neural crest cells that colonize the olfactory nerve. Furthermore, we were unable to detect immunoreactivity for the inner ONL-specific OEC marker neuropeptide tyrosine (NPY; Fig. 3E–F^2^) (Ubink et al., 1994; Ubink and Hökfelt, 2000; Au et al., 2002). Together, these data suggest that in the absence of *Sox10*, neural crest cells colonize the developing olfactory nerve but normal OEC differentiation fails.

### *Sox10* deletion disrupts olfactory axon targeting and olfactory receptor neuron maturation

**Fig. 4.**
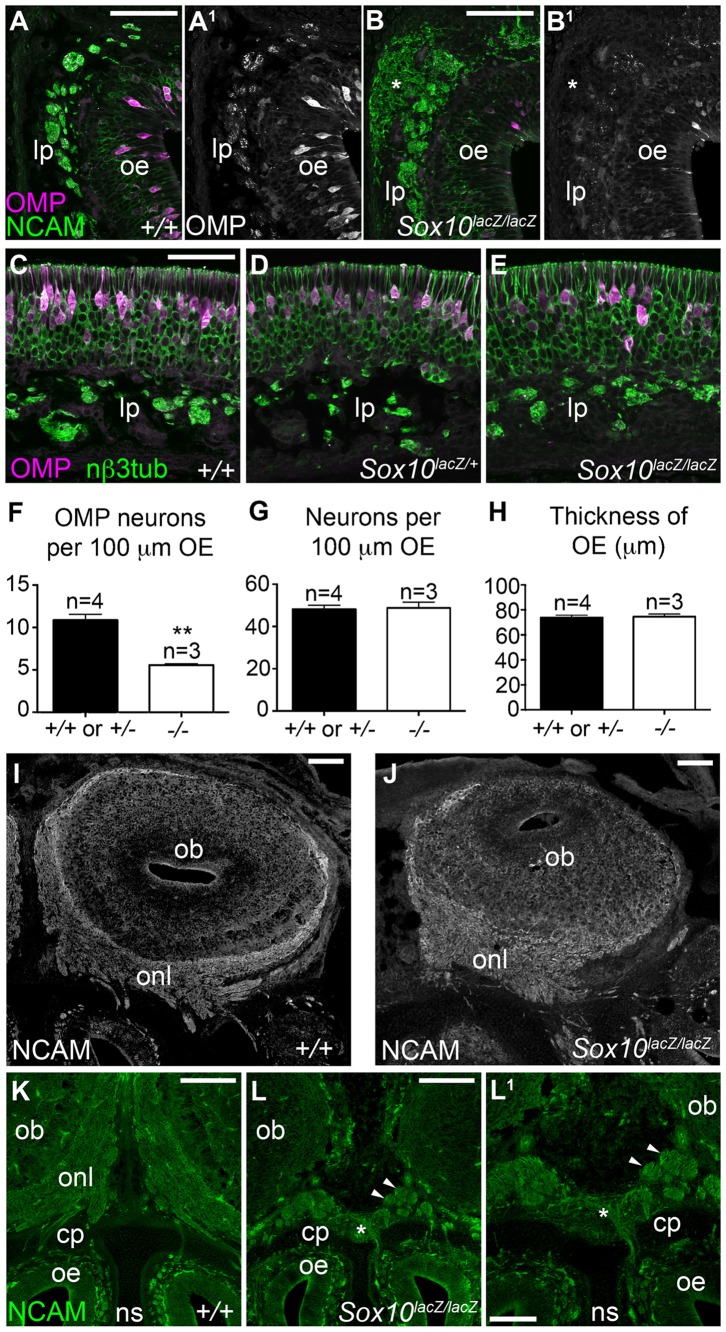
*Sox10* deletion disrupts olfactory axon targeting and ORN maturation. All images show E16.5 coronal sections. (**A–E**) Olfactory mucosa sections immunostained for the maturation marker OMP plus (A–B^1^) NCAM or (C–E) neuronal βIII tubulin. Relative to wild-type (A,A^1^,C) or heterozygote embryos (D), homozygous *Sox10^lacZ/lacZ^* embryos (B,B^1^,E) displayed defasciculated olfactory nerve bundles and inappropriately migrating axons within the lamina propria (asterisk in B,B^1^), and fewer OMP-positive neurons (compare A^1^,B^1^; C–E). (**F–H**) Bar charts showing the mean/embryo + s.e.m. for wild-type/heterozygote embryos (2/genotype) versus homozygous *Sox10^lacZ/lacZ^* embryos, of: (F) OMP-positive cells/100 µm of olfactory epithelium (**P = 0.0014; 2-tailed unpaired t-test); (G) neuronal βIII tubulin-positive cells/100 µm of epithelium; (H) olfactory epithelial thickness. *Sox10* deletion only affects the number of mature (OMP-positive) ORNs. (**I**,**J**) Olfactory bulb sections immunostained for the axonal marker NCAM. Relative to wild-type embryos (I), the ONL in homozygous *Sox10^lacZ/lacZ^* embryos (J) is much thinner in dorsal and lateral regions of the bulb while the ventromedial ONL is much thicker. (**K–L^1^**) NCAM immunostaining showing clearly separated uniform bilateral ONLs in a wild-type embryo (K) versus a merged, ventromedial ONL (asterisk) in a homozygous *Sox10^lacZ/lacZ^* embryo (L,L^1^). Arrowheads in L,L^1^ highlight axonal whorls/balls. Abbreviations as in Fig. 2. Scale bars: 200 µm (K,L), 100 µm (I,J,L^1^), 50 µm (A,B,C).

The absence of lamina propria OECs at E16.5 in homozygous *Sox10^lacZ/lacZ^* embryos was associated with defasciculation of olfactory axon bundles and inappropriate migration of axons within the lamina propria (Fig. 4A–B^1^). We also noticed an apparent reduction in the number of olfactory receptor neurons (ORNs) expressing the maturation marker olfactory marker protein (OMP) (compare Fig. 4A^1^,B^1^; Fig. 4C–E). To investigate this further, we calculated the mean/embryo (± standard error of the mean, s.e.m.) of OMP-positive cells and neuronal βIII tubulin-positive neurons/100 µm of olfactory epithelium, and the thickness of the olfactory epithelium. One-way analysis of variance (ANOVA) using Tukey's multiple comparison test showed no significant difference for any measurement between wild-type (*n* = 2) and heterozygous *Sox10^lacZ/+^* embryos (*n* = 2), so we combined wild-type and heterozygote data (*n* = 4) for comparison with homozygotes (*n* = 3). We confirmed that the mean/embryo (± s.e.m.) of OMP-positive cells/100 µm of epithelium (Fig. 4F) was significantly lower for homozygous *Sox10^lacZ/lacZ^* embryos (5.65±0.17; *n* = 3) than for wild-type/heterozygote embryos (10.86±0.69; *n* = 4) (unpaired 2-tailed t-test: *P* = 0.0014; t = 6.370; 5 degrees of freedom). In contrast, *Sox10* deletion did not affect the mean overall number of neurons/100 µm of epithelium (Fig. 4G: wild-type/heterozygotes: 48.13±1.94; *n* = 4; homozygotes: 48.71±2.82; *n* = 3), or the mean thickness of the olfactory epithelium (Fig. 4H: wild-type/heterozygotes: 73.87±1.91 µm; *n* = 4; homozygotes: 74.60±2.13 µm; *n* = 3), suggesting that *Sox10* deletion specifically affects ORN maturation.

Immunostaining for the axonal marker NCAM also showed that, relative to wild-type, the ONL in dorsal and lateral regions of the olfactory bulb was much thinner after *Sox10* deletion, while the ventromedial ONL was much thicker (Fig. 4I,J). In two homozygous *Sox10^lacZ/lacZ^* embryos, we noticed a ventromedial accumulation of olfactory axons so pronounced that axons from both sides of the nasal cavity merged together ventrally, apparently forming whorls/balls (similar to what is observed in *Gli3^Xt^ extra-toes* mutant mice, which lack olfactory bulbs; St John et al., 2003) rather than a uniform ONL as in wild-type mice (Fig. 4K–L^1^).

These data suggest that the disruption of OEC differentiation arising from *Sox10* deletion results in olfactory axons failing to find their targets in the lateral and dorsal regions of the olfactory bulb, leading to axon accumulation in the ventromedial region and a significant reduction in ORN maturation. When combined with the lack of detectable NPY expression in OECs in the ONL of homozygous *Sox10^lacZ/lacZ^* embryos (Fig. 3E–F^2^), our results are consistent with the previously proposed hypothesis (based on the timing of onset of NPY expression) that NPY secreted from inner-ONL OECs may be involved in the final stages of olfactory axon outgrowth towards glomerular targets (Ubink and Hökfelt, 2000). The effect on maturation is presumably a consequence of defective axon targeting: the maturation marker OMP is only expressed in ORNs that have already contacted the olfactory bulb (Graziadei et al., 1980).

### A significantly smaller proportion of GnRH neurons enters the forebrain after *Sox10* deletion

**Fig. 5.**
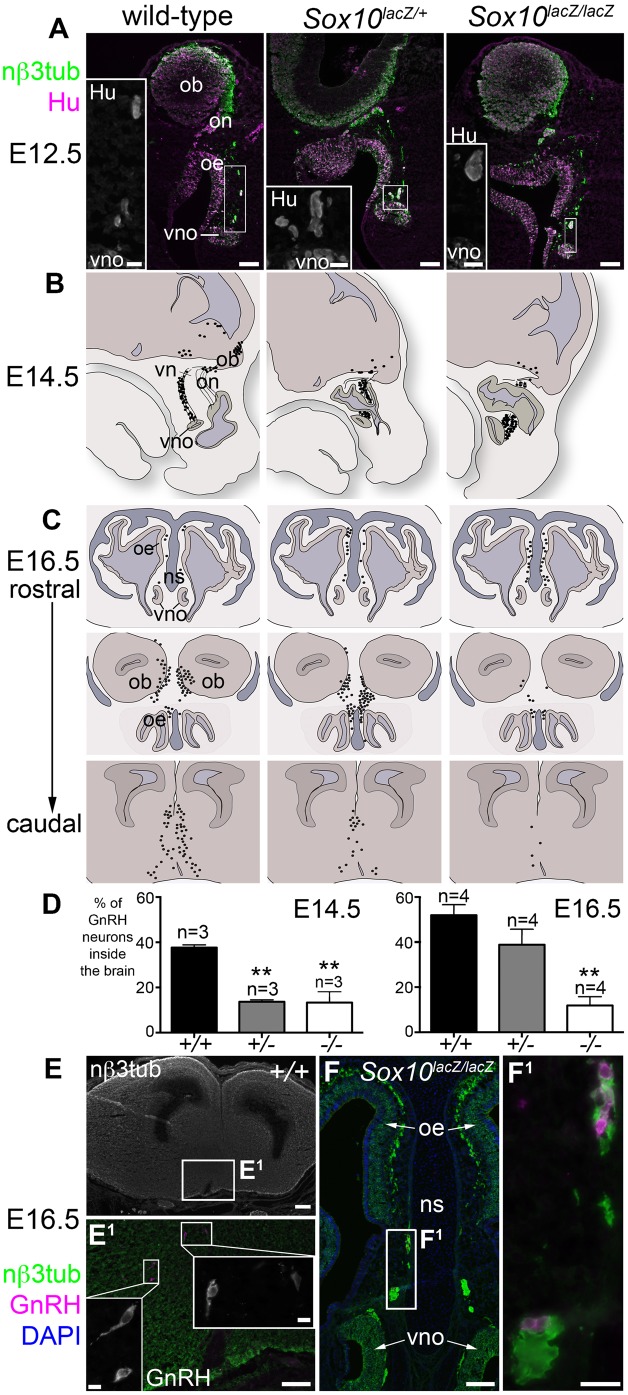
A significantly smaller proportion of GnRH neurons enters the forebrain after *Sox10* deletion. (**A**) At E12.5, immunostaining on coronal sections for neuronal βIII tubulin and the neuronal RNA-binding protein HuC/D revealed unusually large aggregates of multiple neuronal cell bodies on the vomeronasal nerve in both heterozygous *Sox10^lacZ/+^* and homozygous *Sox10^lacZ/lacZ^* embryos. (**B**) Schematic representation of the distribution of GnRH neurons (black spots) at E14.5 on parasagittal sections of 3 wild-type, 3 heterozygous *Sox10^lacZ/+^* and 3 homozygous *Sox10^lacZ/lacZ^* embryos. (**C**) Schematic representation of the distribution of GnRH neurons (black spots) at E16.5 on coronal sections at 3 different rostrocaudal levels of 4 wild-type, 4 heterozygous *Sox10^lacZ/+^* and 4 homozygous *Sox10^lacZ/lacZ^* embryos. (**D**) Bar charts showing the mean percentage/embryo + s.e.m. of GnRH1-positive cells that had entered the brain for wild-type, heterozygous *Sox10^lacZ/+^* and homozygous *Sox10^lacZ/lacZ^* embryos (3 embryos/genotype at E14.5; 4 embryos/genotype at E16.5; ≥100 GnRH neurons counted/embryo). At E14.5, the mean percentage/embryo of GnRH neurons in the brain was significantly lower than wild-type for both heterozygous *Sox10^lacZ/+^* and homozygous *Sox10^lacZ/lacZ^* embryos (**P<0.01; one-way ANOVA using Dunnett's multiple comparison test). By E16.5, the mean percentage/embryo of GnRH neurons in the brain was no longer significantly different from wild-type for heterozygous *Sox10^lacZ/+^* embryos, but was four-fold lower than wild-type for homozygous *Sox10^lacZ/lacZ^* embryos (**P<0.005; one-way ANOVA using Dunnett's multiple comparison test). (**E–F^1^**) Examples of GnRH neurons identified after immunostaining E16.5 coronal sections of wild-type hypothalamus (E,E^1^) and homozygous *Sox10^lacZ/lacZ^* olfactory mucosa (F,F^1^). Abbreviations as in Fig. 2. Scale bars: 200 µm (E), 100 µm (A, main panels; E^1^,F), 25 µm (A, insets; F^1^); 10 µm (E^1^, insets).

Already at E12.5, immunostaining for neuronal βIII tubulin and the neuron cell body-specific Elav RNA-binding protein family members HuC/D (Hinman and Lou, 2008) revealed unusually large aggregates containing multiple neuronal cell bodies on the vomeronasal nerve in both heterozygous *Sox10^lacZ/+^* and homozygous *Sox10^lacZ/lacZ^* embryos (Fig. 5A). This suggested that defective OEC differentiation was affecting the migration of GnRH neurons. The distribution of GnRH neurons in wild-type, heterozygous *Sox10^lacZ/+^* and homozygous *Sox10^lacZ/lacZ^* embryos is illustrated at E14.5 in Fig. 5B (parasagittal sections; GnRH1-positive cells from 3 embryos/genotype, from 3 different litters) and at E16.5 in Fig. 5C (coronal sections at three different rostrocaudal levels; GnRH1-positive cells from 4 embryos/genotype, from 4 different litters). Each black spot represents a GnRH1-positive cell on a photomicrograph, identified either by ISH or immunostaining. We quantified these data by counting ≥100 GnRH1-positive cells/embryo and calculating the mean percentage/embryo ± s.e.m. of GnRH1-positive cells that had entered the brain (Fig. 5D). One-way ANOVA using Dunnett's multiple comparison test showed that at E14.5, the mean percentage/embryo of GnRH neurons that had entered the brain was significantly lower (*P*<0.01) in both heterozygous *Sox10^lacZ/+^* mutants (13.7±0.9%; *n* = 3) and homozygous *Sox10^lacZ/lacZ^* mutants (13.3±4.8%; *n* = 3) than in wild-type embryos (37.7±1.2%; *n* = 3) (Fig. 5D). By E16.5, there was no longer any significant difference between heterozygote (38.8±6.9%; *n* = 4) and wild-type embryos (51.9±4.7%; *n* = 4), but four-fold fewer GnRH neurons were present in the brain in homozygous *Sox10^lacZ/lacZ^* mutants (11.9±3.9%; *n* = 4) than in wild-type embryos (*P*<0.005; also see Fig. 5D). Examples of GnRH neurons in the hypothalamus of wild-type embryos and the olfactory mucosa of homozygous *Sox10^lacZ/lacZ^* mutants are shown in Fig. 5E–F^1^. These data suggest that GnRH neuron migration to the forebrain is delayed when one copy of *Sox10* is missing, and stalled after *Sox10* deletion. A recent report describing a close association between migrating GnRH neurons and OECs (Geller et al., 2013) is consistent with the important role for OECs in GnRH neuron migration that we have demonstrated here.

We conclude that human *Sox10* mutations cause Kallmann's syndrome phenotypes (Bondurand et al., 2007; Barnett et al., 2009) by disrupting the differentiation of OECs, which, as shown here, promote olfactory axon targeting, ORN maturation (most likely because of their importance for olfactory axon targeting) and GnRH neuron migration. A neural crest defect in Kallmann's syndrome is supported by its inclusion within CHARGE syndrome (Pinto et al., 2005), an autosomal dominant disorder caused by heterozygous mutations in *CHD7*, encoding a chromatin-remodeling protein that controls neural crest formation (Bajpai et al., 2010), and by the demonstration that *anosmin1*, loss-of-function mutations in which cause X-linked Kallmann's syndrome (Cadman et al., 2007; Hardelin and Dodé, 2008), promotes cranial neural crest cell formation in an autocrine fashion (Endo et al., 2012). Overall, our results highlight the interplay between neural crest-derived OECs and olfactory placode-derived axons and neurons (Sabado et al., 2012) that seems to be required for both olfaction and fertility.

## Note added in proof

While our manuscript was in revision, another study was published showing that loss-of-function mutations in *SOX10* cause Kallmann's syndrome with deafness and describing the same OEC phenotype in *Sox10* mutant mice, thus implicating neural crest-derived OECs in the aetiology of Kallmann's syndrome (Pingault et al., 2013).

## References

[b1] Au W. W. Treloar H. B. Greer C. A. (2002). Sublaminar organization of the mouse olfactory bulb nerve layer. J. Comp. Neurol. 446, 68–80 10.1002/cne.10182 11920721

[b2] Bajpai R. Chen D. A. Rada-Iglesias A. Zhang J. Xiong Y. Helms J. Chang C. P. Zhao Y. Swigut T. Wysocka J. (2010). CHD7 cooperates with PBAF to control multipotent neural crest formation. Nature 463, 958–962 10.1038/nature08733 20130577 PMC2890258

[b3] Barnett C. P. Mendoza-Londono R. Blaser S. Gillis J. Dupuis L. Levin A. V. Chiang P. W. Spector E. Reardon W. (2009). Aplasia of cochlear nerves and olfactory bulbs in association with *SOX10* mutation. Am. J. Med. Genet. A. 149A, 431–436 10.1002/ajmg.a.32657 19208381

[b4] Barraud P. Seferiadis A. A. Tyson L. D. Zwart M. F. Szabo-Rogers H. L. Ruhrberg C. Liu K. J. Baker C. V. H. (2010). Neural crest origin of olfactory ensheathing glia. Proc. Natl. Acad. Sci. USA 107, 21040–21045 10.1073/pnas.1012248107 21078992 PMC3000254

[b5] Bondurand N. Dastot-Le Moal F. Stanchina L. Collot N. Baral V. Marlin S. Attie-Bitach T. Giurgea I. Skopinski L. Reardon W. (2007). Deletions at the *SOX10* gene locus cause Waardenburg syndrome types 2 and 4. Am. J. Hum. Genet. 81, 1169–1185 10.1086/522090 17999358 PMC2276340

[b6] Britsch S. Goerich D. E. Riethmacher D. Peirano R. I. Rossner M. Nave K. A. Birchmeier C. Wegner M. (2001). The transcription factor Sox10 is a key regulator of peripheral glial development. Genes Dev. 15, 66–78 10.1101/gad.186601 11156606 PMC312607

[b7] Cadman S. M. Kim S.-H. Hu Y. González-Martínez D. Bouloux P.-M. (2007). Molecular pathogenesis of Kallmann's syndrome. Horm. Res. 67, 231–242 10.1159/000098156 17191030

[b8] Cariboni A. Maggi R. Parnavelas J. G. (2007). From nose to fertility: the long migratory journey of gonadotropin-releasing hormone neurons. Trends Neurosci. 30, 638–644 10.1016/j.tins.2007.09.002 17981344

[b9] Cuschieri A. Bannister L. H. (1975). The development of the olfactory mucosa in the mouse: light microscopy. J. Anat. 119, 277–286. 1133096 PMC1231592

[b10] Ekberg J. A. K. Amaya D. Mackay-Sim A. St John J. A. (2012). The migration of olfactory ensheathing cells during development and regeneration. Neurosignals 20, 147–158 10.1159/000330895 22456085

[b11] Endo Y. Ishiwata-Endo H. Yamada K. M. (2012). Extracellular matrix protein anosmin promotes neural crest formation and regulates FGF, BMP, and WNT activities. Dev. Cell 23, 305–316 10.1016/j.devcel.2012.07.006 22898776 PMC3422507

[b12] Finzsch M. Schreiner S. Kichko T. Reeh P. Tamm E. R. Bösl M. R. Meijer D. Wegner M. (2010). *Sox10* is required for Schwann cell identity and progression beyond the immature Schwann cell stage. J. Cell Biol. 189, 701–712 10.1083/jcb.200912142 20457761 PMC2872908

[b13] Forni P. E. Taylor-Burds C. Melvin V. S. Williams T. Wray S. (2011). Neural crest and ectodermal cells intermix in the nasal placode to give rise to GnRH-1 neurons, sensory neurons, and olfactory ensheathing cells. J. Neurosci. 31, 6915–6927 10.1523/JNEUROSCI.6087-10.2011 21543621 PMC3101109

[b14] Geller S. Kolasa E. Tillet Y. Duittoz A. Vaudin P. (2013). Olfactory ensheathing cells form the microenvironment of migrating GnRH-1 neurons during mouse development. Glia 61, 550–566 10.1002/glia.22455 23404564

[b15] Graziadei G. A. M. Stanley R. S. Graziadei P. P. C. (1980). The olfactory marker protein in the olfactory system of the mouse during development. Neuroscience 5, 1239–1252 10.1016/0306-4522(80)90197-9 7402467

[b16] Hardelin J.-P. Dodé C. (2008). The complex genetics of Kallmann syndrome: KAL1, FGFR1, FGF8, PROKR2, PROK2, et al. Sex. Dev. 2, 181–193 10.1159/000152034 18987492

[b17] Henrique D. Adam J. Myat A. Chitnis A. Lewis J. Ish-Horowicz D. (1995). Expression of a *Delta* homologue in prospective neurons in the chick. Nature 375, 787–790 10.1038/375787a0 7596411

[b18] Herbarth B. Pingault V. Bondurand N. Kuhlbrodt K. Hermans-Borgmeyer I. Puliti A. Lemort N. Goossens M. Wegner M. (1998). Mutation of the Sry-related *Sox10* gene in *Dominant megacolon*, a mouse model for human Hirschsprung disease. Proc. Natl. Acad. Sci. USA 95, 5161–5165 10.1073/pnas.95.9.5161 9560246 PMC20231

[b19] Hinman M. N. Lou H. (2008). Diverse molecular functions of Hu proteins. Cell. Mol. Life Sci. 65, 3168–3181 10.1007/s00018-008-8252-6 18581050 PMC2580827

[b20] Lassiter R. N. Dude C. M. Reynolds S. B. Winters N. I. Baker C. V. H. Stark M. R. (2007). Canonical Wnt signaling is required for ophthalmic trigeminal placode cell fate determination and maintenance. Dev. Biol. 308, 392–406 10.1016/j.ydbio.2007.05.032 17604017 PMC3983986

[b21] Miller A. M. Treloar H. B. Greer C. A. (2010). Composition of the migratory mass during development of the olfactory nerve. J. Comp. Neurol. 518, 4825–4841 10.1002/cne.22497 21031554 PMC3682413

[b22] Murdoch B. Roskams A. J. (2007). Olfactory epithelium progenitors: insights from transgenic mice and *in vitro* biology. J. Mol. Histol. 38, 581–599 10.1007/s10735-007-9141-2 17851769

[b23] Paratore C. Eichenberger C. Suter U. Sommer L. (2002). *Sox10* haploinsufficiency affects maintenance of progenitor cells in a mouse model of Hirschsprung disease. Hum. Mol. Genet. 11, 3075–3085 10.1093/hmg/11.24.3075 12417529

[b24] Pingault V. Bodereau V. Baral V. Marcos S. Watanabe Y. Chaoui A. Fouveaut C. Leroy C. Vérier-Mine O. Francannet C. (2013). Loss-of-function mutations in SOX10 cause Kallmann syndrome with deafness. Am. J. Hum. Genet. 92, 707–724 10.1016/j.ajhg.2013.03.024 23643381 PMC3644631

[b25] Pinto G. Abadie V. Mesnage R. Blustajn J. Cabrol S. Amiel J. Hertz-Pannier L. Bertrand A. M. Lyonnet S. Rappaport R. (2005). CHARGE syndrome includes hypogonadotropic hypogonadism and abnormal olfactory bulb development. J. Clin. Endocrinol. Metab. 90, 5621–5626 10.1210/jc.2004-2474 16030162

[b26] Rao M. S. Anderson D. J. (1997). Immortalization and controlled *in vitro* differentiation of murine multipotent neural crest stem cells. J. Neurobiol. 32, 722–746 10.1002/(SICI)1097-4695(19970620)32:7<722::AID-NEU7>3.0.CO;2-6 9183749

[b27] Rozen S. Skaletsky H. (2000). Primer3 on the WWW for general users and for biologist programmers. Methods Mol. Biol. 132, 365–386 10.1385/1-59259-192-2:365 10547847

[b28] Sabado V. Barraud P. Baker C. V. H. Streit A. (2012). Specification of GnRH-1 neurons by antagonistic FGF and retinoic acid signaling. Dev. Biol. 362, 254–262 10.1016/j.ydbio.2011.12.016 22200593 PMC4561506

[b29] Southard-Smith E. M. Kos L. Pavan W. J. (1998). *Sox10* mutation disrupts neural crest development in *Dom* Hirschsprung mouse model. Nat. Genet. 18, 60–64 10.1038/ng0198-60 9425902

[b30] St John J. A. Clarris H. J. McKeown S. Royal S. Key B. (2003). Sorting and convergence of primary olfactory axons are independent of the olfactory bulb. J. Comp. Neurol. 464, 131–140 10.1002/cne.10777 12898607

[b31] Stemple D. L. Anderson D. J. (1992). Isolation of a stem cell for neurons and glia from the mammalian neural crest. Cell 71, 973–985 10.1016/0092-8674(92)90393-Q 1458542

[b32] Ubink R. Hökfelt T. (2000). Expression of neuropeptide Y in olfactory ensheathing cells during prenatal development. J. Comp. Neurol. 423, 13–25 10.1002/1096-9861(20000717)423:1<13::AID-CNE2>3.0.CO;2-P 10861533

[b33] Ubink R. Halasz N. Zhang X. Dagerlind A. Hökfelt T. (1994). Neuropeptide tyrosine is expressed in ensheathing cells around the olfactory nerves in the rat olfactory bulb. Neuroscience 60, 709–726 10.1016/0306-4522(94)90499-5 7936197

[b34] Valverde F. Santacana M. Heredia M. (1992). Formation of an olfactory glomerulus: morphological aspects of development and organization. Neuroscience 49, 255–275 10.1016/0306-4522(92)90094-I 1436469

[b35] Wierman M. E. Kiseljak-Vassiliades K. Tobet S. (2011). Gonadotropin-releasing hormone (GnRH) neuron migration: initiation, maintenance and cessation as critical steps to ensure normal reproductive function. Front. Neuroendocrinol. 32, 43–52 10.1016/j.yfrne.2010.07.005 20650288 PMC3008544

[b36] Wray S. (2010). From nose to brain: development of gonadotrophin-releasing hormone-1 neurones. J. Neuroendocrinol. 22, 743–753 10.1111/j.1365-2826.2010.02034.x 20646175 PMC2919238

[b37] Xu H. Dude C. M. Baker C. V. H. (2008). Fine-grained fate maps for the ophthalmic and maxillomandibular trigeminal placodes in the chick embryo. Dev. Biol. 317, 174–186 10.1016/j.ydbio.2008.02.012 18367162

